# Geographically weighted generalized poisson regression model with the best kernel function in the case of the number of postpartum maternal mortality in east java

**DOI:** 10.1016/j.mex.2023.102002

**Published:** 2023-01-05

**Authors:** Sischa Wahyuning Tyas, Gunardi, Lila Aryani Puspitasari

**Affiliations:** Department of Mathematics, Faculty of Mathematics and Natural Sciences, Universitas Gadjah Mada, Indonesia

**Keywords:** Poisson distribution, GPR, GWGPR, Bandwidth, Kernel, Cross-validation (CV), MMR, Geographically Weighted Generalized Poisson Regression (GWGPR)

## Abstract

This study proposes a Geographically Weighted Generalized Poisson Regression (GWGPR) model with the best kernel function to obtain a model of the number of postpartum maternal mortality in East Java Province in 2020 and determine the factors that affect the number of maternal postpartum mortality in East Java in 2020. The kernel functions used in this study are fixed bisquare kernel, fixed tricube kernel, and adaptive bisquare kernel. Optimum bandwidth selection using the Cross-Validation (CV) method. The results obtained the best model is the GWGPR model with a fixed bisquare kernel because it produces the smallest AIC value of 194.92. Variables significantly affecting the number of maternal postpartum mortality in East Java in 2020 vary in each district/city where there are three regional groups. The percentage of pregnant women who had a pregnancy visit K1, the percentage of pregnant women who had a pregnancy visit K4, the percentage of households receiving cash assistance, and the ratio of hospitals and health centres have a significant effect on Kabupaten Blitar, Mojokerto, Gresik, Bangkalan, Blitar City, Mojokerto City, Surabaya City. While the five predictor variables together significantly affect districts/cities included in group 3, such as Ponorogo, Trenggalek, Tulungagung, Kediri, Malang, Lumajang, Jember, Banyuwangi, Bondowoso and so on. Some of the highlights of the proposed approach are:

• Generalized Poisson regression model using the maximum likelihood estimation (MLE) method.
• The kernel functions used in the Geographically Weighted Generalized Poisson Regression (GWGPR) model to determine bandwidth are fixed bisquare, fixed tricube, and adaptive bisquare kernel functions selected using the Cross Validation (CV) method.
• The computation procedure is easy to implement.

## Graphical abstract

**Figure:**
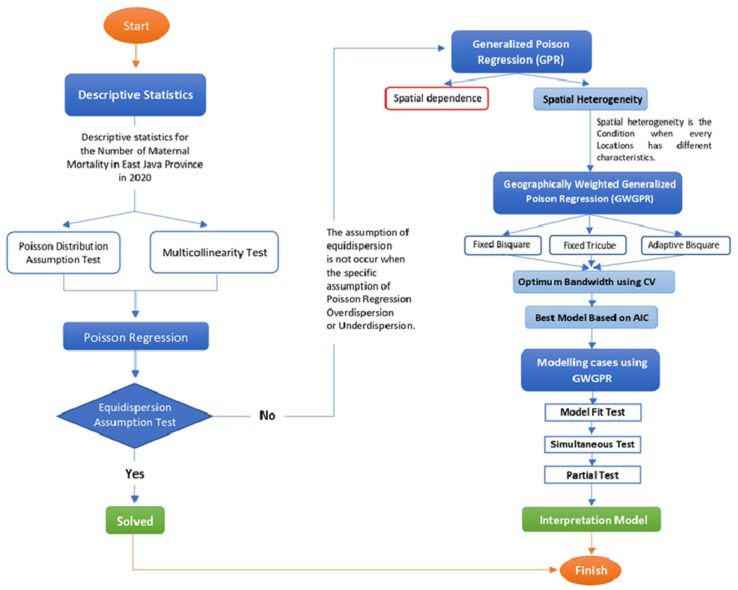

Specifications table

**Table:** 

Subject area:	Mathematics and Statistics
More specific subject area:	Statistics: Regression, Spatial Regression.
Name of your method:	Geographically Weighted Generalized Poisson Regression (GWGPR)
Name and reference of the original method:	**Original Method** Geographically weighted Poisson regression models with different kernels **Reference** Murakami D., Tsutsumida N., Yoshida T., “Stable Geographically Weighted Poisson Regression for Count Data, GIScience 2021 Short Paper Proceedings. (2021).
Resource availability:	Several maternal postpartum mortalities (Y) from East Java Province Health Profile 2020 and the predictors (X) from the Central Statistics Agency in East Java.

## Method details

### Introduction

Regression analysis is a statistical method used to determine the relationship between variables. In a study, it is often found that the object of research is in the form of count data influenced by several explanatory variables. As the case of the number of maternal deaths is a rare event with discrete data, the appropriate regression analysis model to explain the relationship between the dependent variable in the form of discrete and rare data with independent variables in the form of discrete, continuous, categorical, or mixed data is the Poisson regression model. The Poisson regression model is a non-linear regression model used to model count or discrete data, with the main requirement being that the response variable has a Poisson distribution [1]. In the Poisson regression model, there is a specific assumption that the mean and variance of the response variable are equal (equidispersion).

However, there are times when the equidispersion assumption is only sometimes met. Namely, here can also be cases of overdispersion (the value of the data variance is greater than the value of the mean) or underdispersion (the value of the data variance is smaller than the value of the mean) in data modeling with a Poisson distribution [2]. One method that can be used to overcome these cases is Generalized Poisson Regression (GPR). The generalized regression model is one of the alternative models counting data with overdispersion or underdispersion cases in Poisson regression. Generalized Poisson Regression modeling produces a global regression model for all observation locations. In some instances, each observation location has a set of data that is different from one location to another, so to overcome this diversity, spatial data analysis can be used. Spatial regression modeling is performed on response variables in the form of count data that experience underdispersion or overdispersion and depend on the characteristics of the observed location using Geographically Weighted Generalized Poisson Regression (GWGPR). The GWGPR model develops generalized Poisson Regression by considering weights in the form of latitude and longitude of the observation location points. The GWGPR model's parameter estimator is local for each point or observation location. Some research on the GWGPR model is research by Ghanim, Al-Hasani, et al. (2021) entitled "Geographically weighted Poisson regression models with different kernels: application to road traffic accidents," analysing traffic accident data in the country of Oman using the Geographically Weighted Generalized Poisson Regression method with optimum weighting using adaptive kernel functions, namely box-car, bi-square, tricube, exponential and Gaussian [3].

Murakami, Daisuke et al. (2021) used the Geographically Weighted Generalized Poisson Regression method to analyze the covid-19 case in Tokyo, Japan. In this study, the bandwidth used was an adaptive and fixed Gaussian kernel [4]. Maternal Mortality Rate (MMR) is the number of women who die from a cause of death related to pregnancy disorders or their handling during pregnancy, childbirth, or in the maternal postpartum period (42 days after childbirth) but not caused by accidents or injuries [5]. MMR is an essential indicator in determining the degree of community welfare, especially in the health sector. MMR in developing countries such as Indonesia is still relatively high. Based on data from the Ministry of Health in 2020, maternal mortality in Indonesia reached 4627. This figure increased by 10.25% compared to 2019 [6]. East Java Province ranks second in the region with the highest MMR in Indonesia, with 565 maternal mortalities, most of which are caused by maternal postpartum mortality, amounting to 284 people [7]. In their research, Fronczak et al. (2005) stated that 75% of complications occurred postpartum, resulting in high maternal mortality [8]. This is an essential concern for the Indonesian government, especially in East Java Province, so MMR is one of the Sustainable Development Goals (SDGs) targets from 2015 to 2030, namely 70 per 100,000 live births in 2030 [9]. To reduce MMR, problems related to pregnancy, childbirth, and especially the postpartum period (42 days after delivery) cannot be separated from the various factors that influence it. This research will examine case data on maternal postpartum mortality in East Java in 2020 using the GWGPR method. In the GWGPR model, the optimum weight selection uses the fixed kernel bisquare, fixed kernel tricube and adaptive kernel bisquare functions, which are selected using the Cross-Validation (CV) method. Researchers use this method because there has been no previous research using the three kernels mentioned and the indicators considered to affect the dependent variable are also new.

## Model specifications and estimation procedures

### Multicollinearity

Multicollinearity in the regression model can be determined using the Variance Inflation Factor (VIF), which is expressed in the following equation:

(1) $$\text{VI}F_{j}=\frac{1}{1-R_{j}^{2}}$$

where $R_{j}^{2}=\frac{\text{SSR}}{SST}=\frac{\sum_{i=1}^{n}{\left({\hat{X}}_{ij\;}-\;{\bar{X}}_{ij}\right)}^{2}\;\;}{\sum_{i=1}^{n}{\left(X_{ij\;}-\;{\bar{X}}_{ij}\right)}^{2}},$ SSR (Sum of Squares Regression) is the variation caused by the relationship between predictor variables, SST (Sum of Squares Total) is a measure of the variation of the value of X from the mean of X itself, $X_{ij\;}$ is the value of $X_{j\;}$ at the ith observation, $\;{\bar{X}}_{j}$ is average value of $X_{j\;}$ and ${\hat{X}}_{j\;}$ is the predicted value of $X_{j\;}$ at ith observation.

### Poisson distribution

In some research, it is often found that the object of research is in the form of count data influenced by several explanatory variables. A regression model based on the Poisson distribution can determine the relationship pattern between variables. The Poisson distribution is a discrete distribution that measures the probability of a certain number of events or events occurring within a certain period. The probability of a Poisson distribution with a random variable Y and parameter μ>0, which represents the number of successes that occur in a given time interval or region, is [10]

(2) $$f\left(Y;\;\mu \right)=\frac{e^{-\mu \;}\mu {\;}^{y}}{y!}y=1,\;2,\;3,\;\ldots$$

where μ is the average number of ’successes’ that occur in a given time interval or region and e=2.7183. The Poisson distribution has the same mean value and variance as μ.

### Poisson regression

The Poisson regression model is a non-linear regression model used to model count or discrete data, with the main requirement being that the response variable has a Poisson distribution. Poisson regression modeling the expected value of the response variable, with E(Y) as a linear function of the predictor variables $\left(X_{i\;}\right)$ is defined as follows [11].

(3) $$E\;\left(Y\;|\;X_{i\;}\right)=\;\mu \;\left(X_{i\;},\;\beta \right)$$

where

$$X_{i}^{T}=\left[\begin{array}{ccccc} 1 & x_{i1} & x_{i2} & \cdots & x_{ik} \end{array}\right]$$

$$\beta \;={\left[\begin{array}{ccccc} \beta_{0} & \beta_{1} & \beta_{2} & \cdots & \beta_{k} \end{array}\right]}^{T}$$

Since the mean (μ) of Poisson distribution is always positive, the function $\mu (X_{i\;},\;\beta )$ is chosen such that the linear predictor is:

(4) $$\eta_{i}\;=\;\beta_{0}+\;\beta_{1}x_{i1}+\;\beta_{2}x_{i2}+.\;.\;.+\;\beta_{k}x_{ik}$$

which can represent any real value to a positive real value. The logarithmic function is a suitable *link* function to model Poisson regression in the Generalized Linear Model approach, is:

(5) $$\text{ln}\;E\left(Y|X_{i\;}\right)=\ln\mu_{i}=\beta_{0}+\beta_{1}x_{i1}+\beta_{2}x_{i2}+.\;.\;.+\beta_{k}x_{ik}=\eta_{i}$$

or

(6) $$\mu_{i}=\exp\left(\beta_{0}+\beta_{1}x_{i1}+\beta_{2}x_{i2}+.\;.\;.+\beta_{k}x_{ik}\right)=\exp\left(X_{i}^{T}\beta \right)$$

Thus, the Poisson regression model for the response variable Y, which is Poisson distributed with parameter $\mu_{i}$ is:

(7) $$\mu_{i}=\exp\left(X_{i}^{T}\beta \right)$$

Parameter estimation of the Poisson regression model can be done using the Maximum Likelihood Estimation (MLE) method. The maximum likelihood estimator for the parameter (β) is expressed by $\left(\hat{\beta }\right)$, which is obtained by maximizing the likelihood function. Assuming that $y_{1},y_{2}$,…, $y_{n}$ are random variables that are mutually independent and $y_{n}∼\text{Poisson}\;\left(\mu_{i}\right),\;$then the likelihood function for Poisson regression model is:

(8) $$L\left(\beta \right)=\prod_{i=1}^{n}f\left(y_{i};\beta ,\mu_{i}\right)\;=\left[\frac{\left[\prod_{i=1}^{n}\mu_{i}^{yi}\right]\text{exp}\left[-\sum_{i=1}^{n}\mu_{i}\right]}{\prod_{i=1}^{n}y_{i}!}\right]$$

The log-likelihood function of the Poisson regression model is:

$$\ln L\left(\beta \right)=\left[\frac{\left[\prod_{i=1}^{n}\mu_{i}^{yi}]\text{exp}[-\sum_{i=1}^{n}\mu_{i}\right]}{\prod_{i=1}^{n}y_{i}!}\right]\;=\ln\left[\prod_{i=1}^{n}\mu_{i}^{yi}\right]\text{ln}\;\text{exp}\;\left[-\sum_{i=1}^{n}\mu_{i}\right]--\;\text{ln}\;\prod_{i=1}^{n}y_{i}!=\sum_{i=1}^{n}y_{i}\text{ln}\mu_{i}-\;\sum_{i=1}^{n}\mu_{i}-\sum_{i=1}^{n}y_{i}!$$

Because of $\mu_{i}=\exp\left(X_{i}^{T}\beta \right)$ so that

(9) $$\ln L\left(\beta \right)=\sum_{i=1}^{n}y_{i}\ln\left[\exp\left(X_{i}^{T}\beta \right)\right]-\;\sum_{i=1}^{n}\exp\left(X_{i}^{T}\beta \right)-\;\sum_{i=1}^{n}\ln y_{i}!\;=\sum_{i=1}^{n}y_{i}\left(X_{i}^{T}\beta \right)-\;\sum_{i=1}^{n}\left(X_{i}^{T}\beta \right)-\;\sum_{i=1}^{n}\text{ln}\;y_{i}!$$

Furthermore, to obtain the estimator $(\hat{\beta }),$ the log-likelihood function in Eq. (9) ismaximized by lowering it concerning the parameter β and equating it to zero. Parameter estimation using the MLE method obtained an equation that is not closed form, so we can estimate the parameter $\hat{\beta }$ by using the Newton-Raphson iteration method as follows:

(10) $$equal{\hat{\beta }}_{\left(m+1\right)}={\hat{\beta }}_{\left(m\right)}-H^{-1}\left(\beta_{m}\right)\;g\left(\beta_{m}\right)\;$$

where $H^{-1}\left(\beta_{m}\right)$ is Hessian matrix and $g(\beta_{m})$ is gradient vector.

Testing the parameters of the Poisson regression model is carried out to test whether the regression model parameters have a significant effect on the response variable (Y). Model parameter testing using the partial and simultaneous tests is as follows.

Hypothesis:

$H_{0}$$:\beta_{1}=\;\beta_{2}=\ldots =\;\beta_{k}=0$
$H_{1}$: at least one $\beta_{p}$ ≠ 0, *p* = 1, 2, …, k

The test statistic used:

(11) $$D\left(\hat{\beta }\right)=-2\;\text{ln}\left(\frac{L\left(\hat{w}\right)}{L\;\left(\hat{\Omega }\right)}\right)=-2\left(\text{ln}\;L\left(\hat{w}\right)-L\;\left(\hat{\Omega }\right)\right)\;$$

The $L(\hat{w})$ function is the maximum likelihood value for a simple model with no predictors involved, and $L\;(\hat{\Omega })$ is the maximum likelihood value for a complete model with predictor variables involved. The rejection region of $H_{0}$ is to reject $H_{0}$ if the value of $D\left(\hat{\beta }\right)>\;x_{\left(a,k\right)}^{2}$, this means that there is at least one variable that has a significant influence on the response variable (Y). The smaller the value of $D(\hat{\beta })$ indicates, the smaller the error rate in the model [12].

After testing the parameters simultaneously, we will continue with partial parameter testing. The partial parameter testing hypothesis is as follows:

Hypothesis:

$H_{0\;}$$:\beta_{p}=0,\;p=1,\;2,\;\ldots ,\;k$
$H_{1\;}$:$\beta_{p}$ ≠ 0,

The test statistic used:

(12) $$Z=\frac{{\hat{\beta }}_{p}}{se{\hat{\beta }}_{p}}$$

The rejection region of $H_{0\;}$ is to reject $H_{0\;}$ at the significance level α if the value of $|Z|>Z_{\left(\alpha /2\right)}$ this means that the variable *p* has a significant effect on the response variable.

### Overdispersion or underdispersion in poisson regression models

The presence of overdispersion or underdispersion in Poisson regression can be detected using the devians, and *Pearson Chi-Square* values divided by the degrees of freedom. A value or quotient more significant than 1 indicates the presence of overdispersion $(\text{var}\left(Y_{i}|X_{i}^{T}\right)>E\left(Y_{i}|X_{i}^{T}\right)),$ whereas if the result of dividing the two values is smaller than 1 indicating the presence of under dispersion $\text{var}\left(Y_{i}|X_{i}^{T}\right)>E\left(Y_{i}|X_{i}^{T}\right)$, then one method that can be used to overcome this case is Generalized Poisson Regression [13].

### Generalized poisson regression

The Generalized Poisson Regression (GPR) model is used for counting data that experience equidispersion violations. The Generalized Poisson Regression model has the same form as Poisson Regression as follows:

(13) $$\mu_{i}=\exp\left(X_{i}^{T}\beta \right)$$

In the GPR model, the values of the parameters $\phi ,\beta_{0},\;\beta_{1},\beta_{2},\;\ldots ,\;\beta_{k}$ will be estimated using the Maximum Likelihood Estimation (MLE) method with the likelihood

The function of the GPR model are as follows:

(14) $$L\;\left(\mu_{i},\;\phi \right)=\;\prod_{i=1}^{n}{\left(\frac{\mu_{i}}{1+\phi \mu_{i}}\right)}^{y_{i}}\frac{{\left(1+\phi y_{i}\right)}^{y_{i}-1}}{y_{i}!}\text{exp}\left(-\frac{\mu_{i}\left(1+\phi y_{i}\right)}{1+\phi \mu_{i}}\right)$$

Next, Newton Raphson iteration is performed to maximize the log-likelihood function formulated as follows:

$$\ln L\left(\phi ,\;\beta \right)=\sum_{i=1}^{n}\left[y_{i}\left(X_{i}^{T}\beta -\ln(1+\phi \exp\left(X_{i}^{T}\beta \right)\right)\right]+\left(y_{i}-1\right)\ln\left(1+\phi y_{i}\right)-\ln y_{i}!-\frac{\exp\left(X_{i}^{T}\beta \right)\left(1+\phi y_{i}\right)}{1+\phi \exp\left(X_{i}^{T}\beta \right)}$$

Perform Newton Raphson iteration based on equation:

(15) $${\hat{\beta }}_{\left(m+1\right)}={\hat{\beta }}_{\left(m\right)}-H^{-1}\left(\beta_{m}\right)\;g\;\left(\beta_{m}\right)$$

The iteration process will stop if it has found an estimated value that converges to a value as follows:

(16) $${\hat{\beta }}_{\left(m+1\right)}\approx {\hat{\beta }}_{\left(m\right)}\;{\hat{\beta }}_{\left(m+1\right)}-{\hat{\beta }}_{\left(m\right)}\langle \varepsilon ,\varepsilon \rangle 0$$

Furthermore, the estimation of the parameter ϕ using the Newton-Raphson iteration method is:

(17) $$\phi {\;}_{\left(m+1\right)}=\phi {\;}_{\left(m\right)}-{\left(\frac{\partial^{2}\text{lnL}\left(\mu_{i},\phi {\;}_{\left(m\right)}\right)}{\partial \phi^{2}}\right)}^{-1}\left(\frac{\partial \text{lnL}\left(\mu_{i},\phi {\;}_{\left(m\right)}\right)}{\partial \phi }\right)$$

A more accessible approach to estimating the parameter phi is to use the estimation of moments, equating the Pearson Chi-Square statistic with the degrees of freedom [14]. To determine ϕ with the method of moments can be expressed by the equation:

(18) $$\sum_{i=1}^{n}\frac{y_{i}-\mu_{i}}{\text{var}\left(Y_{i}\right)}-\left(n-k\right)=0$$

Thus, the following iteration is obtained:

(19) $$\phi {\;}_{\left(m+1\right)}=\phi {\;}_{\left(m\right)}-{\left(-2\sum_{i-1}^{n}\frac{{\left(y_{i}-\mu_{i}\right)}^{2}}{{\left(1+\phi \mu_{i}\right)}^{3}}\right)}^{-1}\left(\sum_{i=1}^{n}\frac{{\left(y_{i}-\mu_{i}\right)}^{2}}{\mu_{i}{\left(1+\phi \mu_{i}\right)}^{2}}\right)-\left(n-k\right)$$

where $\mu_{i}=\text{exp}\left(X_{i}^{T}\beta \right)>0$

GPR parameter testing is done with the Maximum Likelihood Ratio Test (MLRT) with the following hypothesis:

$H_{0}$$:\beta_{1}=\;\beta_{2}=\ldots =\;\beta_{k}=0$
$H_{1}$: at least one $\beta_{p}$ ≠ 0, *p* = 1, 2, …, k

The test statistic used:

$$D\left(\hat{\beta }\right)=-2\text{ln}\left(\frac{L\left(\hat{w}\right)}{L\;\left(\hat{\Omega }\right)}\right)=-2\left(\text{ln}\;L\left(\hat{w}\right)-L\;\left(\Omega \right)\right)$$

The $H_{0}$ rejection region is to reject $H_{0}$ if the value of$\;D\left(\hat{\beta }\right)>x_{\left(a,k\right)}^{2}$, this means that there is at least one variable that has a significant effect on the model. If the simultaneous test decision is to reject $H_{0}$, then the next step is to test the parameters partially to find out which parameters have a significant effect on the model. The hypothesis used is as follows:

$H_{0\;}$$:\beta_{p}=0,\;p=1,\;2,\;\ldots ,\;k$
$H_{1\;}$:$\beta_{p}$ ≠ 0,

The test statistic used:

$$W=\frac{{\hat{\beta }}_{p}}{se{\hat{\beta }}_{p}}$$

The rejection region of $H_{0\;}$ is to reject $H_{0\;}$ at the significance level α if the value of $|W|>Z_{\left(\alpha /2\right)}$ this means that the variable *p* has a significant effect on the response variable.

### Spatial heterogeneity test

Spatial heterogeneity occurs when the same predictor variable has an unequal effect on different locations within a study area. Spatial heterogeneity can be tested using the Breusch-Pagan (BP) test. Hypotheses used in the Breusch- Pagan test is as follows.

$H_{0\;}$$:\;\sigma_{1}^{2}=\sigma_{2}^{2}=\ldots =\sigma_{n}^{2}=\sigma^{2}$ (Variance between locations is equal)
$H_{1}$: at least there is one $\sigma_{1}^{2}\neq \sigma^{2}$ (Variance between locations is different)
  (20) $$\text{BP}=\frac{1}{2}f^{T}Z{\left(Z^{T}Z\right)}^{-1}Z^{T}f$$

Where the vector element $f={\left(f_{1},f_{2},\;\ldots ,\;f_{n}\right)}^{T}\;$ is $f_{1}=\frac{e_{i}^{2}}{{\hat{\sigma }}^{2}}$ with $e_{i}=y_{i}-\hat{y_{i}}$, Z is a matrix of size n×(k+1) containing the vector of normalized X for each other ${\hat{\sigma }}^{2}$ is the residual variance ($e_{i}$). Reject $H_{0\;}$at significance level α if the value of $\text{BP}>X_{\left(\alpha ,k\right)}^{2}$or p−value<α, this means that the variance between locations is different.

### Geographically weighted regression

Geographically weighted regression is used to analyze spatial heterogeneity, where each parameter is calculated at each observation location, so each region or observation location has different regression parameter values. Systematically the Geographically Weighted Regression (GWR) model with response variable *y* and predictor variables $x_{1},x_{2},\;\ldots ,\;x_{k}$at the ith location can be written as follows:

(21) $$y_{i}=\beta_{0}\left(u_{i},v_{i}\right)+\sum_{p=1}^{k}\beta_{p}\left(u_{i},v_{i}\right)x_{ip}+\varepsilon_{i},\;i=1,\;2,\;\ldots ,\;n\;$$

In spatial analysis, the role of bandwidth for GWR models is essential because the weight value represents the location of observations between one data and another. There are three types of bandwidth functions used in this study:

i. Fixed Bisquare

The form of fixed bisquare is expressed by the following formula:

(22) $$w_{ij}=\left\{ \begin{array}{c} {\left[1-{\left(\frac{d_{ij}}{h}\right)}^{2}\right]}^{2}\;for\;d_{ij}<h\\ 0,\;for\;others \end{array}\right.\;$$

ii. Fixed Tricube

The following formula expresses the form of the fixed tricube:

(23) $$w_{ij}=\{\begin{array}{c} {\left[1-{\left(\frac{d_{ij}}{h}\right)}^{3}\right]}^{3}\;for\;d_{ij}<h\\ 0,\;for\;others \end{array}\;$$

iii. Adaptive Bisquare
  (24) $$w_{ij}=\left\{ \begin{array}{c} {\left[1-\left(\frac{d_{ij}}{h_{i}}\right){}^{2}\right]}^{2}\;for\;d_{ij}<h\\ 0,\;for\;others \end{array}\right.\;$$

with $h_{i}$ is the bandwidth at the th observation location.

One of the methods used to determine the optimum bandwidth size is the Cross-Validation (CV) method. CV method is defined as follows:

(25) $$\text{CV}=\sum_{i=1}^{n}{\left(Y_{i}-{\hat{Y}}_{\neq i}\left(h\right)\right)}^{2}\;$$

where ${\hat{Y}}_{\neq i}\left(h\right)$ is the value of the estimator $Y_{i\;}$ for observations at locations $u_{i},v_{i}$ not included in the calculation. The optimum bandwidth value (ℎ) is obtained when ℎ produces the minimum CV value.

### Geographically weighted generalized poisson regression

Spatial regression modeling is performed on response variables in the form of count data that experience underdispersion or overdispersion and depend on the characteristics of the observed location using Geographically Weighted Generalized Poisson Regression. The difference between global Poisson regression and GWGPR is in parameter estimation. The global Poisson regression model has the same parameter estimator value for each observation location, so it is global. The GWGPR model produces a different parameter estimator value for each observation location area, so it is local [15].

The GWGPR model is the development of Generalized Pois-son Regression by considering the weights in the form of latitude and longitude of the observation location points. The GWGPR model follows the distribution of Generalized Poisson Regression so that the probability function for each ith location is as follows:

(26) $$f_{i}\left(y_{i},u_{i},\phi \right)={\left(\frac{u_{i}}{1+\phi u_{i}}\right)}^{y_{i}}\frac{{\left(1+\phi y_{i}\right)}^{y_{i}-1}}{y_{i}!}\text{exp}\left(-\frac{u_{i}\left(1+\phi y_{i}\right)}{1+\phi u_{i}}\right)$$

GWGPR modeling uses a generalized linear model with a “g” function (link function) that connects the mean of the response variable with the linear predictor (η). The link function used in the GWGPR model is the log link. The GWGPR model with $u_{i}$ as the latitude coordinate and as the longitude coordinate used as the parameter weight is

(27) $$\begin{array}{cc} \eta_{i} & =g\;\left(u_{i}\right)=\beta_{0}\left(u_{i},v_{i}\right)+\beta_{1}\left(u_{i},v_{i}\right)x_{i1}+\ldots +\beta_{k}\left(u_{i},v_{i}\right)x_{ik}\;\eta_{i}=g\left(\mu_{i}\right)=\ln\left(\mu_{i}\right)=X_{i}^{T}\beta \left(u_{i},v_{i}\right)\mu_{i}\\ & =g^{-1}\left(\eta_{i}\right)=g^{-1}X_{i}^{T}\beta \left(u_{i},v_{i}\right)\;\mu_{i}=\text{exp}\left(\beta_{0}\left(u_{i},v_{i}\right)+\beta_{1}\left(u_{i},v_{i}\right)x_{i1}+\ldots +\beta_{k}\left(u_{i},v_{i}\right)x_{ik}\right) \end{array}$$

### Parameter estimation of GWGPR model

In the GWGPR model, the method used to estimate the model parameters is the Maximum Likelihood Estimation (MLE) method, with the GWGPR model likelihood function as follows:

(28) $$L\left(\beta \left(u_{i},v_{i}\right)\right)\;=\prod_{i=1}^{n}f\left(y_{i}\right)=\prod_{i=1}^{n}{\left(\frac{u_{i}}{1+\phi u_{i}}\right)}^{y_{i}}\frac{{\left(1+\phi u_{i}\right)}^{y_{i}-1}}{y_{i}!}\text{exp}\left(-\frac{u_{i}\left(1+\phi y_{i}\right)}{1+\phi u_{i}}\right)$$

Furthermore, the likelihood function in Eq. (28) is con-verted into natural logarithm form as follows:

(29) $$\begin{array}{cc} \ln L\left(\beta \left(u_{i},v_{i}\right),\phi \right)\; & =\sum_{i=1}^{n}\text{ln}\left[{\left(\frac{u_{i}}{1+\phi u_{i}}\right)}^{y_{i}}\frac{{\left(1+\phi y_{i}\right)}^{y_{i}-1}}{y_{i}!}\exp\left(-\frac{u_{i}\left(1+\phi y_{i}\right)}{1+\phi u_{i}}\right)\right]\\ & =\sum_{i=1}^{n}\left[y_{i}\left(\text{ln}\;u_{i}-\text{ln}\left(1+\phi u_{i}\right)\right)+\left(y_{i}-1\right)\ln\left(1+\phi y_{i}\right)-\text{ln}\;y_{i}!-\frac{u_{i}\left(1+\phi y_{i}\right)}{1+\phi u_{i}}\right] \end{array}$$

By substituting the value $\mu ={\text{exp}\;X}_{i}^{T}\beta \left(u_{i},v_{i}\right)$ the Eq. (30) is obtained.

(30) $$L\left(\beta \left(u_{i},v_{i}\right)\phi \right)=\sum_{i=1}^{n}\left[y_{i}\left(\text{ln}e^{X_{i}^{T}\beta \left(u_{i},v_{i}\right)}-\text{ln}\left(1+\phi e^{X_{i}^{T}\beta \left(u_{i},v_{i}\right)}\right)\right)\;\right]+\left(y_{i}-1\right)\text{ln}\left(1+\phi y_{i}\right)-\text{ln}\;y_{i}!-\frac{e^{X_{i}^{T}\beta \left(u_{i},v_{i}\right)}\left(1+\phi y_{i}\right)}{1+\phi e^{X_{i}^{T}\beta \left(u_{i},v_{i}\right)}}$$

The process of obtaining parameter estimators of the GWGPR model is by deriving the log-likelihood function for each parameter and then equating it to zero. Because the results are not close form, it is necessary to do a Newton-Raphson iteration with the following algorithm.

1. Determine the initial estimated values of the parameters
  $$\hat{\beta }{\left(u_{i},v_{i}\right)}_{\left(0\right)}=\left[\phi_{0}\left(u_{i},v_{i}\right)\;\beta_{0}\left(u_{i},v_{i}\right)\;\beta_{10}\left(u_{i},v_{i}\right)\ldots \;\beta_{k0}\left(u_{i},v_{i}\right)\right]$$
2. Form a gradient vector (**g**) with *k* estimated parameters
  $$g^{T}{\left(\beta {\left(u_{i},v_{i}\right)}_{\left(m\right)}\right)}_{\left(k+1\right)\times \;1}={\left(\frac{\partial \text{lnL}\left(\beta \left(u_{i},v_{i}\right)\right)}{\partial \phi \left(u_{i},v_{i}\right)},\frac{\partial \text{lnL}\left(\beta \left(u_{i},v_{i}\right)\right)}{\partial \beta_{0}\left(u_{i},v_{i}\right)},\frac{\partial \text{lnL}\left(\beta \left(u_{i},v_{i}\right)\right)}{\partial \beta_{1}\left(u_{i},v_{i}\right)},\ldots ,\frac{\partial \text{lnL}\left(\beta \left(u_{i},v_{i}\right)\right)}{\partial \beta_{k}\left(u_{i},v_{i}\right)}\right)}_{\beta =\beta_{\left(m\right)}}$$
3. Form the Hessian matrix (H) which is the second derivative of Eq. (30)
4. Substitute the values of $\hat{\beta }{\left(u_{i},v_{i}\right)}_{\left(0\right)}$ into the elements of the g vector and H matrix to obtain the gradient vector $g(\hat{\beta }{\left(u_{i},v_{i}\right)}_{\left(0\right)})$ and Hessian matrix $H(\hat{\beta }{\left(u_{i},v_{i}\right)}_{\left(0\right)})$**.**
5. Perform iteration using equation:
  $$\hat{\beta }{\left(u_{i},v_{i}\right)}_{(m+1)}=\hat{\beta }{\left(u_{i},v_{i}\right)}_{\left(m\right)}-H^{-1}\left(\hat{\beta }{\left(u_{i},v_{i}\right)}_{\left(m\right)}\right)g\left(\hat{\beta }{\left(u_{i},v_{i}\right)}_{\left(m\right)}\right)$$
6. The iteration process starts from *m* = 0, and the value of $\hat{\beta }{\left(u_{i},v_{i}\right)}_{\left(m\right)}\;$ is a set of parameter estimators that converge at the mth iteration for the ith location.
7. If you have not gotten a convergent parameter estimation, then the iteration continues back to step 5 until iteration m=m+1. The iteration process is stopped if
  $$|\hat{\beta }{\left(u_{i},v_{i}\right)}_{(m+1)}-\hat{\beta }{\left(u_{i},v_{i}\right)}_{\left(m\right)}|\leq \varepsilon$$

where ε is a very small number.

### Simultaneous parameter significance test

The method used for simultaneous parameter testing in the GWGPR model is the Maximum Likelihood Ratio Test (MLRT) method. Testing the parameters of the GWGPR model is carried out to determine the significance of the β parameter with the following hypothesis.

$H_{0}:\beta_{1}\left(u_{i},v_{i}\right)=\beta_{2}\left(u_{i},v_{i}\right)=\ldots =\beta_{k}\left(u_{i},v_{i}\right)$
$H_{1}$: at least one$\;\beta_{p}\left(u_{i},v_{i}\right)\neq 0,\;p=1,\;2,\;\ldots ,k$

Test Statistic:

$$D\left(\hat{\beta }\right)=-2\text{ln}\left(\frac{L\left(\hat{w}\right)}{L\;\left(\hat{\Omega }\right)}\right)=-2\left(\text{ln}\;L\left(\hat{w}\right)-L\;\left(\Omega \right)\right)$$

$D(\hat{\beta })$ is the devians value of GWGPR model, $w=\{\beta_{0}\}$ is the set of parameters under $H_{0}\;\text{and}\;\Omega =\beta_{0},\beta_{1},\beta_{2},\ldots ,\beta_{k}$ is the set of parameters under population.$\;L(\hat{w})$ is the maximum likelihood value for a simple model without involving predictors, andL(Ω) is the maximum likelihood value for the complete model involving variables Furthermore, the value of D$\left(\hat{\beta }\right)$ is obtained by solving equation as follows:

$$D\left(\hat{\beta }\right)=-2\left(\text{ln}\;L\left(\hat{w}\right)-L\;\left(\Omega \right)\right)$$

$D(\hat{\beta })$ is $x^{2}$ distributed with k independent degrees. The decision to reject $H_{0}$ if the value of $D\left(\hat{\beta }\right)>x_{\left(a,k\right)}^{2}$means that there is at least one variable that has a significant effect on the response variable.

### Partial parameter significance test

Partial GWGPR model parameter testing is conducted to determine the effect of individual predictor variables on the response variable at each location. The partial parameter testing hypothesis is as follows.

$H_{0}:\beta_{p}\left(u_{i},v_{i}\right)=0,p=1,\;2,\ldots ,\;k$
$H_{1}$$:\beta_{p}\left(u_{i},v_{i}\right)\neq 0$

The Statistics:

$$Z=\frac{{\hat{\beta }}_{p}\left(u_{i},v_{i}\right)}{se\left({\hat{\beta }}_{p}\left(u_{i},v_{i}\right)\right)}$$

where $se({\hat{\beta }}_{p}\left(u_{i},v_{i}\right))$ is the standard error of ${\hat{\beta }}_{p}\left(u_{i},v_{i}\right)$. The $H_{0}$ rejection area is to reject $H_{0}\;\text{at}\;\text{the}\;\alpha$ significance level if the value $|Z|>Z_{\left(\alpha /2\right)}$ which means that variable p has a significant effect on the response variable at each location in the GWGPR model.

### Akaike information criterion

Akaike Information Criterion (AIC) is one of the criteria to determine the best model. A small AIC value indicates that the model is getting better. The best model selection using the AIC value criterion is as follows:

AIC=−2lnL(β)+2k

is maximum log-likelihood model and k is number of parameters estimated in the model.

## Data

### Maternal postpartum mortality rate

Maternal Mortality Rate (MMR) is the number of female mortalities that occur during pregnancy or its handling during pregnancy, childbirth, or within 42 days after the end of preg nancy (postpartum period) but not mortality caused by accidents or injuries. MMR is an essential indicator in determining the welfare of society, especially in the health sector. The Ministry of Health in 2020 revealed that the number of maternal mortalities in Indonesia is still relatively high, reaching 4627 people. East Java Province ranks second with the number of maternal postpartum mortality at 284. The number of maternal postpartum mortality in East Java Province over five years is presented in the following figure.

Several studies have found that the factor that has a significant effect on the number of maternal postpartum mortality is the percentage of pregnant women who had a pregnancy visit K4 [16]. Furthermore, there is research on the number of maternal postpartum mortality, with one of the factors affecting maternal mortality is the percentage of handling obstetric complications in each district/city [17]. Another study on the number of maternal postpartum mortality found that one of the factors affecting maternal postpartum mortality was the percentage of pregnant women who had a pregnancy visit K1 [18]. There is also research on factors that are thought to affect maternal mortality such as the percentage of households receiving cash assistance and the ratio of health centers and hospitals [19].

Based on the description above, in this study, several variables are used that are thought to affect the number of maternal postpartum mortality in East Java in 2020. Y is the number of maternal postpartum mortality, $x_{1}$ is the percentage of pregnant women who had a pregnancy visit K1, $x_{2}$ is the percentage of obstetric complications, $x_{3}$ is the percentage of pregnant women who had a pregnancy visit K4, $x_{4}$ is percentage of households receiving cash assistance, and $x_{5}$ is ratio of health centers and hospital.

### Characteristics of maternal postpartum mortality rate in east java

Descriptive analysis was carried out to obtain the characteristics of the number of maternal postpartum mortality (Y) and the factors that influence it. In 2020 the number of maternal postpartum mortality in East Java was 284. Descriptive statistics of all variables used and presented in Table 1 below.

**Table 1:** Overview of Maternal Postpartum Mortality Rate in East Java and Factors Suspected To Affect It.

Variables	Min	Max	Mean	Variance
Y	1	30	7.47	31.553
X_1_	86.60	105.90	98.2184	16.479
X_2_	0	25.80	7.6579	73.464
X_3_	78.50	99.30	90.2763	25.641
X_4_	4.14	28.21	13.2650	30.216
X_5_	4.48	17.98	7.1982	6.717

Table 1. shows that the factors that are thought to affect the number of maternal postpartum mortality in East Java have a large enough variance and a large enough range that data heterogeneity is suspected. The predictor variable with the highest variance is the percentage of obstetric complications $\left(x_{2}\right)$ of 73.464%. The percentage of pregnant women who had a preg nancy visit K1 $\left(x_{1}\right)$ in East Java had an average of 98.22% with the highest percentage in Kabupaten Bondowoso at 105.9% and the lowest percentage in Nganjuk District at 86.6%. The handling of obstetric complications $\left(x_{2}\right)$ in East Java is quite good. This can be seen from the average value of the percentage of obstetric complications of 7.657% with the lowest percentage of complications in Kabupaten Trenggalek, Blitar, Lumajang, Jember, Bondowoso, Situbondo, Probolinggo, Mo jokerto, Jombang, Magetan, Ngawi, Bojonegoro, Kediri City, Probolinggo City, while the highest percentage was in Pasuruan city at 25.8%. The average percentage of pregnant women who had a pregnancy visit K4 was 90.28%, with the highest rate in Madiun City at 99.3% and the lowest percentage in Kabupaten Situbondo at 78.5%. The percentage of households receiving cash assistance was lowest in Surabaya City at 4.14%. The ratio of health centers and hospitals has an average of 7.2, with the lowest ratio being Kabupaten Malang at 4.48 while the highest percentage is Mojokerto City at 17.98.

An overview of the distribution of cases of the number of maternal postpartum mortality in East Java in 2020 is presented in Fig. 2.

## Results and analysis

### Multicollinearity test

One of the requirements that must be met in Poisson regression analysis is multicollinearity detection. The presence of multicollinearity can be known from each predictor variable's VIF (Variance Inflation Factor) value. A VIF value of more than 10 indicates a case of multicollinearity.

Table 2 shows that the VIF value for all predictor variables is less than ten, so there are no cases of multicollinearity in the data.

**Table 2:** Independent variable VIF value.

Variable	VIF
X_1_	1.68
X_2_	1.16
X_3_	1.33
X_4_	1.53
X_5_	1.20

### Distribution fit test

The distribution suitability test is used to determine whether the data is Poisson distributed or not. To conduct the test, you can use the Kolmogorov Smirnov Test with the following hypothesis:

H_0_ : Data follows Poisson distribution
H_1_ : Data does not follow Poisson distribution Significance level: α=0.05

Test Statistic

$$D=\text{max}1<i<N\;\left(F\left(Y_{i}\right)-\frac{i-1}{N},\frac{i-1}{N}-F\left(Y_{i}\right)\right)$$

where F $\left(Y_{i}\right)$ is the cumulative probability distribution. Test criteria: accept $H_{0}$ if the value of $D<D_{N,\alpha }$ in the Kolmogorov Smirnov table or the >α Based on the output, the p−value=0.087 is obtained, so with a significance level of α=0.05, then the p−value=0.087>0.05, this means that the data on the number of maternal postpartum mortality is Poisson distributed.

### Spatial heterogeneity test and the best bandwidth

Spatial heterogeneity testing is conducted to determine whether there are characteristics between observation location points. Spatial heterogeneity can be tested using the Breusch- Pagan (BP) test with the following hypothesis test:

$H_{0}:\;\sigma_{1}^{2}=\sigma_{2}^{2}=\ldots =\sigma_{38}^{2}=\sigma^{2}$ (Variance between locations is the same)
$H_{1}:$ there is at least one $\sigma_{i}^{2}\neq \sigma^{2};i=1,\;2,\;\ldots ,\;38$ (Variance between locations is the different)

The test statistic value is 17.802 and the p−value is 0.003206. The significance level used is 5%, so $x_{0.05;5}^{2}\;$ is obtained at 11.07. Therefore, it is concluded that reject $H_{0}$, or the variance between locations is different. This means that there is spatial diversity between regions, or the characteristics between location points are different.

The existence of spatial effects causes spatial heterogeneity, so spatial weighting is needed. The best spatial weighting is obtained from the minimum Cross-Validation (CV) value's bandwidth value. Table 3 shows the optimum bandwidth selection value using the fixed bisquare, fixed tricube and adaptive bi-square kernel functions.

**Table 3:** Optimum bandwidth selection.

Kernel Function	CV
Fixed Bisquare	1367.024
Fixed Tricube	1377.01
Adaptive Bisquare	1401.472

Table 3 shows that the kernel function that produces the optimum bandwidth is the fixed bisquare Kernel function with a CV value of 1367.024. After obtaining the optimum bandwidth value, the spatial weight matrix for each observation location will be obtained by substituting the bandwidth value and Euclid distance.

### Model testing

#### Poisson regression

Based on the result, obtaining the estimated value of the Poisson regression model parameters can be done using the Maximum Likelihood Estimation (MLE) method with Newton- Raphson iterations. The estimated values of the Poisson regression model parameters are presented in Table 4 below.

**Table 4:** Parameter estimation of poisson regression model.

Parameter	Estimated value	SE	Z	P-value
β_0_	5.527718	1.563	3.535	0.000408
β_1_	0.012778	0.017647	0.724	0.469008
β_2_	−0.02061	0.007963	−2.588	0.00966
β_3_	−0.0253	0.013211	−1.915	0.055458
β_4_	−0.04565	0.013695	−3.333	0.000859
β_5_	−0.25964	0.040695	−6.38	0.000000000177
Devians	72,026			
AIC	225,19			

Table 4 shows that the AIC value of the Poisson regression model is 225.19. The predictor variables that have a significant effect on the Poisson regression model are the percentage of handling obstetric complications $\left(x_{1}\right)$, the percentage of households receiving cash assistance $\left(x_{4}\right)$ and the ratio of health centers and hospitals $\left(x_{5}\right)$. So that the effect of predictor variables $x_{1},x_{4},x_{5}$ is significant but the effect of predictor variables $x_{2},x_{3}$ is not significant on the response variable. The Poisson regression model formed in the case of the number of maternal postpartum mortality in East Java Province in 2020 is

$$\hat{\mu }=\exp\left(5.5277+0.01278x_{1}-\;0.0206x_{2}-0.025x_{3}\;-\;0.04565x_{4}\;-\;0.25964x_{5}\right)$$

#### Generalized poisson regression

From the Poisson regression modeling, it is known that the data on the number of maternal postpartum mortality experience overdispersion cases because D$\;(\hat{\beta })$ value of the Poisson regression model in Table 4 is 72.026 with an independent degree of 32, resulting in a value of 2.25. This value is greater than 1, so the method used to overcome these cases is Generalized Poisson Regression (GPR). The Maximum Likelihood Estimation (MLE) method is used with Newton-Raphson iterations to obtain the estimated value of the GPR model parameters. Table 4 shows the estimated values of the GPR model parameters.

Based on Table 5, it is known that the values of devians D $\left(\hat{\beta }\right)$ is 202.4872. The significance level used is 0.05, so the value of $x_{0.05;5}^{2}$ is 11.07. The devians value D $\left(\hat{\beta }\right)$ is more than $x_{0.05;5}^{2}$which means reject $H_{0}$. So it can be concluded that at least one predictor variable has a significant effect on the model.

**Table 5:** Parameter estimation of generalized poisson regression model.

Parameter	Estimated value	SE	Z	P-value
β_0_	5.460	2.154	2.535	0.0113
β_1_	0.009827	0.024482	0.401	0.6881
β_2_	−0.01925	0.010911	−1.764	0.0777
β_3_	−0.02257	0.018373	−1.229	0.2192
β_4_	−0.04172	0.018785	−2.221	0,0264
β_5_	−0.25167	0.054851	−4.588	0.00000447
Devians	202.4872			
AIC	216.4872			

The partial parameter test results show that the variables that have a significant effect are the percentage of households receiving cash assistance $\left(x_{4}\right)$ and the ratio of health centers and hospitals $\left(x_{5}\right)$ so that the GPR model for the case of the number of maternal mortalities in East Java Province in 2020 is as follows.

$$\hat{\mu }\;=\;\text{exp}\;(5.4603+0.0098x_{1}-\;0.019247x_{2}-0.02257x_{3}\;-\;0.04172x_{4}\;-\;0.2516x_{5})$$

#### Geographically weighted generalized poisson regression

Testing the parameters of the GWGPR model is carried out to determine the significance of the parameters β with the following hypothesis**:**

$H_{0}:\;\beta_{1}\left(u_{i},v_{i}\right)=\beta_{2}\left(u_{i},v_{i}\right)=\ldots =\beta_{2}\left(u_{i},v_{i}\right)=0$
$H_{1}:$ there is at least one $\beta_{p}\left(u_{i},v_{i}\right)\neq 0,\;p=1,\;2,\;\ldots ,\;5$

Based on the result, the devians value D $\left(\hat{\beta }\right)$ is 180.9181. The significance level used is 5%, so $x_{0.05;5}^{2}$ is 11.07. The devians value of D$\left(\hat{\beta }\right)$ is greater than the value of $x_{0.05;5}^{2}$ so the test decision is to reject $H_{0}$. This means that at least one predictor variable has a significant effect on the GWGPR model. Furthermore, the significance of the GWGPR model parameters is partially carried out to determine which predictor variables or factors have a significant effect in each region or location. The partial parameter testing hypothesis is as follows:

$H_{0}:\;\beta_{p}\left(u_{i},v_{i}\right)=0,\;i=1,\;2,\ldots ,\;38,\;p=1,\;2,\;\ldots ,\;5$
$H_{1}:\beta_{p}\left(u_{i},v_{i}\right)\neq 0$

The predictor variable is said to have a significant effect on the response variable if the value of

$|Z|>Z_{\left(\alpha /2\right)}$. The significance level used is 5%, so the $Z_{\left(\alpha /2\right)}$ value is 1.96. Based on the result, the percentage of pregnant women who had a pregnancy visit K1 $\left(x_{1}\right)$, the percentage of pregnant women who had a pregnancy visit K4 $\left(x_{3}\right)$ and the percentage of households receiving cash assistance $\left(x_{4}\right)$ had a significant effect on 38 districts/cities. While the percentage of obstetric complications $(x_{2})\;$has a significant effect on 31 districts/cities and the ratio of health centers and hospitals $\left(x_{5}\right)$ has a significant effect on 37 districts/cities. The significant variables in each district/city in East Java are presented as follows:

• Variable $x_{1},x_{2},\;x_{3},\;x_{4},x_{5}$ significant to 1 district (Kabupaten Pacitan).
• Variable $x_{1},x_{2},\;x_{3},\;x_{4},x_{5}$ significant to 7 district/city (Kabupaten Blitar, Mojokerto, Gresik, Bangkalan, Blitar city, Mojokerto city, Surabaya city).
• Variable $x_{1},x_{2},\;x_{3},\;x_{4},x_{5}$ significant to 30 district/city (Kabupaten Ponorogo, Trenggalek, Tulungagung, Kediri, Malang, Lumajang, Jember, Banyuwangi, Bondowoso, Situbondo, Probolinggo, Pasuruan, Sidoarjo, Jombang, Nganjuk, Madiun, Magetan, Ngawi, Bojonegoro, Tuban, Lamongan, Sam-pang, Pamekasan, Sumenep, Kediri city, Malang city, Probolinggo city, Pasuruan city, Madiun city, Batu city).

The grouping of districts/cities in East Java based on significant variables is presented in Fig. 3.

Based partial parameter testing, as an example, we will present parameter testing at the 3rd research location $(u_{3},v_{3})=$, namely Kabupaten Trenggalek, with parameter estimates shown in Table 6.

**Table 6:** Parameter testing of GWGPR model in kabupaten trenggalek with fixed bisquare kernel.

Parameter	Estimated Value	Z count
β_0_	4.02379	−0.00618
β_1_	−0.00183	−2503.51
β_2_	−0.01013	7.150146
β_3_	−0.00583	1.146733
β_4_	−0.01475	6.524979
β_5_	−0.14298	3.770794
Devians	180.92	
AIC	194.92	

Table 6 shows that all predictor variables have a significant effect on the GWGPR model in Trenggalek District because the $Z_{count}$ value is greater than $Z_{0.05/2}$. The GWGPR model formed in the case of the number of maternal postpartum mortality in the Kabupaten Trenggalek is as follows.

$$\hat{\mu }\;=\;\text{exp}\;(4.023\;-\;0.0018x_{1}\;-\;0.01013x_{2}-0.0058x_{3}\;-\;0.0417x_{4}\;-\;0.1429x_{5})$$

Based on the GWGPR model in Kabupaten Trenggalek, every 5% increase in the percentage of pregnant women who had a pregnancy visit K1 will reduce the average number of maternal postpartum mortality in East Java by exp0.00183=0.998 times, assuming other variables are constant. Suppose the percentage of pregnant women who had a pregnancy visit K4 increases by 5%. In that case, it will reduce the aver- age number of maternal postpartum mortality in East Java by exp(−0.00583)=0.994 times, assuming other variables are constant. The same interpretation applies to the predictor variables of the percentage of obstetric complications, the percent- age of households receiving cash assistance also variable the ratio of health centers and hospitals.

##### Determination of the best model

Based on Table 4, it is known that for the Poisson regression model, three variables have a significant effect on the model, namely the percentage of obstetric complications $\left(x_{2}\right)$, the per- centage of households receiving cash assistance $\left(x_{4}\right)$ also the ratio of hospitals and health centers $\left(x_{5}\right)$.

Table 5 shows that the variables that significantly affect the GPR model are the percentage of households receiving cash assistance $\left(x_{4}\right)$ and the ratio of hospitals and health, centers $\left(x_{5}\right)$. Meanwhile, in the GWGPR model, the variables of the percentage of pregnant women who had a pregnancy visit K1$\left(x_{1}\right)$, the percentage of pregnant women who had a pregnancy visit k4 $\left(x_{3}\right)$, and the percentage of households receiving cash assistance $\left(x_{4}\right)$ together influence 38 districts/cities. Further- more, the variables of obstetric complications and the ratio of hospitals and health centers have different influences for each district/city.

One of the criteria for determining the best model is to look at the Akaike Information Criterion (AIC) value obtained. The smaller the AIC value, the better the model used. The following are the AIC values for the Poisson, GPR and GWGPR regression models.

**Fig. 1:**
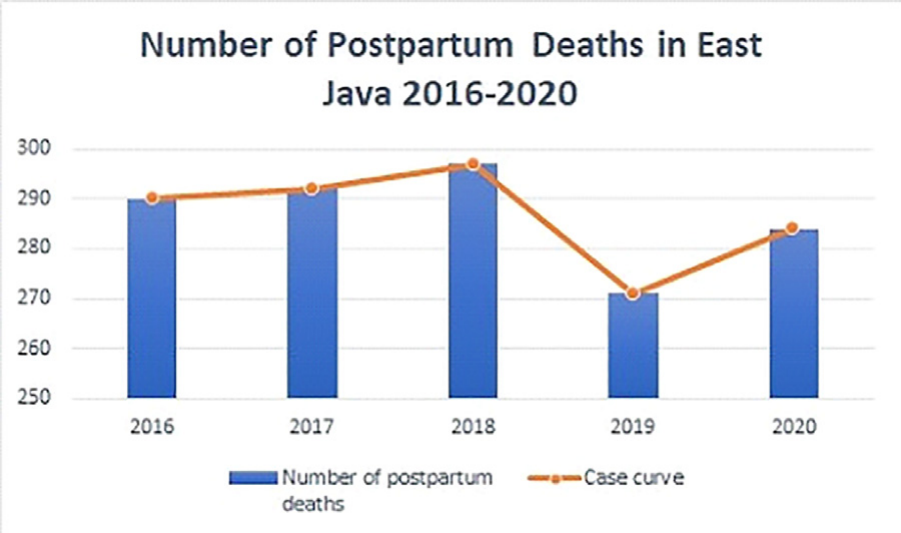
Maternal Postpartum Mortality Rate in East Java 2016–2020.

The AIC value of the Poisson regression model is 225.19, the AIC value of the GPR model is 216.4872, and the GWGPR model with the best kernel (fixed bisquare) has an AIC value of 194.92. This means that the GWGPR model is the best because it has the smallest AIC value compared to other models (Algorithm 1, Fig. 1, Table 7).

**Algorithm 1:** Algorithm 1 is the procedure for estimating the parameters of the GWGPR model in the case of the number of maternal deaths in East Java in 2020.

1. Researchers are looking for case data on the number of maternal postpartum mortality in East Java Province in 2020 and longitude also latitude coordinate data.
2. Analyzing the Poisson regression model
3. Performing generalized Poisson regression model analysis
4. Analyze the Geographically Weighted Generalized Pois- son Regression (GWGPR) model with the following steps:
a. Determine ($u_{i},v_{i}$) based on longitude and latitude for each district.
b. Breusch-Pagan test to see the presence of spatial heterogeneity in the data.
c. Calculating the Euclidean distance between observation locations based on geographical position.
d. Determining the optimum bandwidth using the Cross- Validation (CV) method.
e. Calculate the best weight matrix using fixed bisquare kernel, tricube kernel and adaptive bisquare kernel.
f. Parameter estimation of the GWGPR model using the Maximum Likelihood Estimation (MLE) method followed by Newton-Raphson iteration.
g. Simultaneous and partial significance testing of model parameters
h. Interpreting the GWGPR model obtained.
i. Determining the best model using the AIC value, the best model is the model with the smallest AIC value.
5. The final result of this research is to get the best model to represent the number of maternal postpartum mortality and know the mapping of the number of maternal postpartum mortality in East Java Province.

**Fig. 2:**
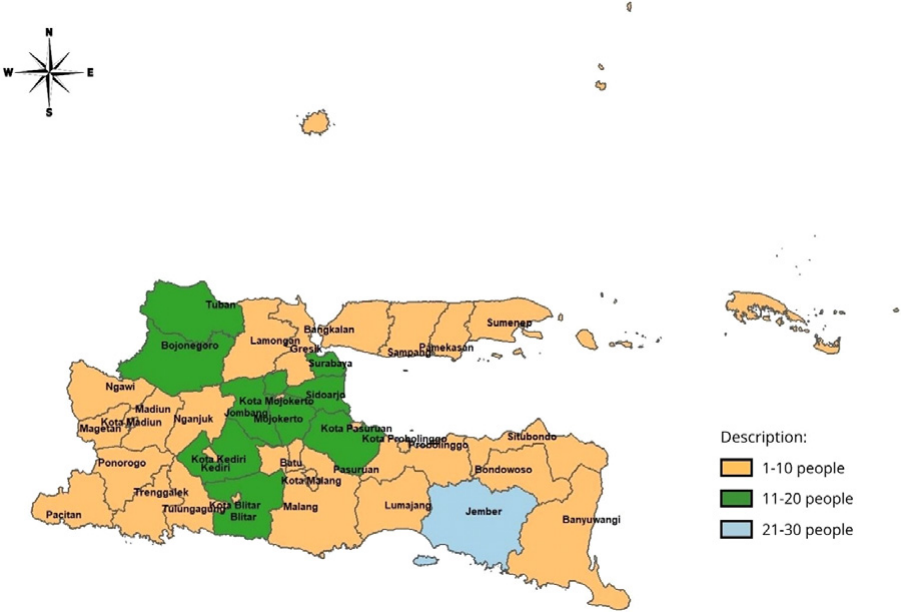
Maternal Postpartum Mortality Rate in East Java 2020.

**Fig. 3:**
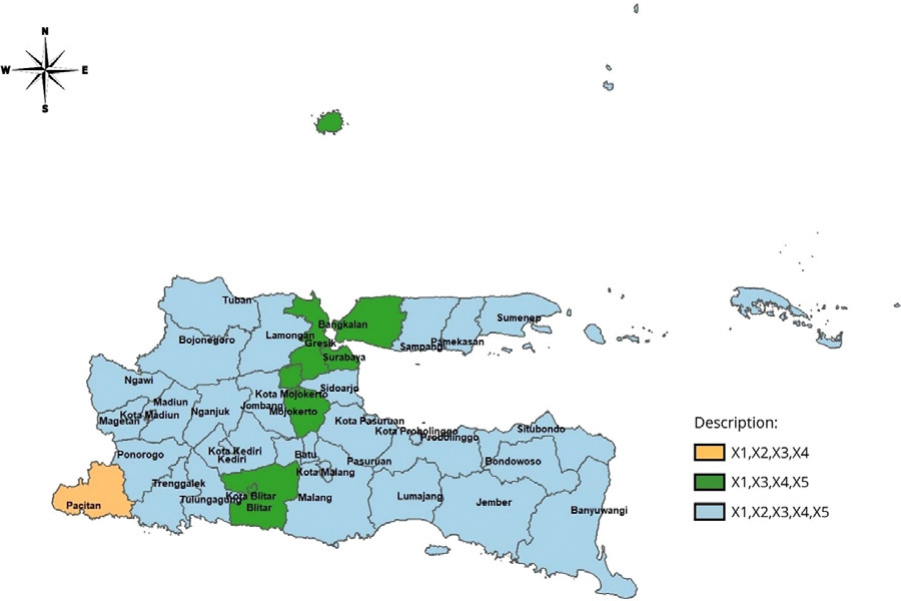
Mapping Based on Significant Variables.

**Table 7:** Best model with AIC value.

Regression Model	AIC
Poisson Regression	225.19
Generalized Poisson Regression	216.4872
Geographically Weighted Generalized Poisson Regression	194.92

## Conclusion

The average number of maternal postpartum mortality in each district/city in East Java Province was 7.47 in 2020, with the highest death of 30 cases in Kabupaten Jember and the lowest case in Sampang, Kediri city, Madiun City and Batu city. The high variance value of 31.553 indicates a reasonably high difference in maternal postpartum mortality in each district/city. The best kernel function is the fixed bisqure kernel function with a CV value of 1367.024. Based on the results obtained, the GWGPR model is the best model to model the case of the number of maternal postpartum mortality in East Java in 2020 because it produces the smallest AIC value of 194.92 with three groupings obtained based on the results of partial testing for GWGPR modeling.

## Ethics statements

The data used in this study are secondary data derived from the 2020 East Java Provincial Health Profile published by the East Java Provincial Health Office and some secondary data derived from the official website of the Badan Pusat Statistik (BPS) East Java.

## CRediT authorship contribution statement

**Ghanim Al-Hasani:** Methodology, GWPR Methods; **Murakami, Daisuke:** Spatial, Validity tests; **Gunardi:** Conceptualization, Conceptual.

## Declaration of Competing Interest

The authors declare that they have no known competing financial interests or personal relationships that could have appeared to influence the work reported in this paper.

## Data Availability

Data will be made available on request.
